# Differential expression of *hsa-miR-221, hsa-miR-21, hsa-miR-135b,* and *hsa-miR-29c* suggests a field effect in oral cancer

**DOI:** 10.1186/s12885-018-4631-z

**Published:** 2018-07-06

**Authors:** Camile B. Lopes, Leandro L. Magalhães, Carolina R. Teófilo, Ana Paula N. N. Alves, Raquel C. Montenegro, Massimo Negrini, Ândrea Ribeiro-dos-Santos

**Affiliations:** 1 Laboratory of Human and Medical Genetics, Graduate Program of Genetics and Molecular Biology, Federal University of Pará, Belém, PA 66075-110 Brazil; 2 Department of Clinical Dentistry - Health Sciences Center, Federal University of Ceará, Fortaleza, CE 60020-181 Brazil; 3 Center of Research and Drug Development, Federal University of Ceara, Fortaleza, CE 60430-270 Brazil; 4 Department of Morphology, Surgery and Experimental Medicine, University of Ferrara, 44121 Ferrara, Italy; 5 Research Center of Oncology, Federal University of Pará, 66, Belém, PA 073-005 Brazil

**Keywords:** Oral cancer, Field cancerization, miRNAs, Biomarkers

## Abstract

**Background:**

The theory of field effect suggests that the tumor-adjacent area, besides histopathologically normal, undergoes genetic and epigenetic changes that can eventually affect epithelial homeostasis, predisposing the patient to cancer development. One of the many molecular changes described in cancer are microRNAs (miRNAs), which regulates the expression of important genes during carcinogenesis. Thus, the aim of this study was to investigate the field effect in oral cancer.

**Methods:**

We investigated the differential expression profile of four miRNAs (*hsa-miR-221*, *hsa-miR-21*, *hsa-miR-135b*, and *hsa-miR-29c*) in cancerous oral tissue, in tumor-adjacent tissue and and in non-cancerous tissue samples from healthy volunteers.

**Results:**

Our results showed significant overexpression profiles of all four studied miRNAs in cancerous oral tissue compared to non-cancerous samples, as well as in tumor-adjacent tissue compared to cancer-free tissue. No significant difference was found when comparing the expression profile of cancerous and tissue-adjacent tissue groups. We found a negative correlation between the expression of *hsa-miR-21* expression and STAT3 in oral squamous cell carcinoma.

**Conclusion:**

These results suggest that the tissue adjacent to cancer cannot be considered a normal tissue because its molecular aspects are significantly altered. Our data corroborates the hypothesis of field cancerization.

## Background

Oral cancer is the most common type of head and neck cancer and includes lesions in the lips and in the oral cavity (buccal mucosa, hard palate, floor of the mouth, tongue, gums, retromolar trigone, and alveolar ridge). Oral squamous cell carcinoma (OSCC) represents 90% of the neoplasms in the oral cavity and is characterized by an aggressive and invasive growth pattern that spreads to the cervical lymph nodes. In most cases, OSCC has a mutilating characteristic and causes irreversible consequences to speech, breathing and swallowing. This affects the health and self-image of the patient, which can result in its social isolation. Therefore, it is a traumatic type of malignancy and causes a significant impact on the patient’s life quality [1–3].

Additionally, OSCC has a high mortality rate, mainly due to cervical lymph node metastasis, locoregional recurrence, and distant metastases in the lungs and bones [4, 5].

The theory of field effect or field cancerization, firstly described by Slaughter in 1953, shows that the tumor-adjacent area, besides histopathologically normal, undergoes genetic and epigenetic changes that can eventually lead to the development of local recurrence or onset of a second primary tumor [6].

Changes in the post-transcriptional regulation of mRNAs by microRNA (miRNAs) activity play an important role in carcinogenesis [7–9]. Mature miRNAs molecules are small non-coding single-stranded RNA molecules (18–25 nucleotides) [10, 11]. They are involved in several regulatory pathways, including cell development, differentiation, proliferation, aging, senescence and apoptosis. Deregulation of miRNA expression contributes to the manifestation of several diseases, including cancer [12–14].

The expression profile of several miRNAs is tissue-specific [15, 16]. Thus, the comparative analysis of miRNAs expression between tissues with and without cancer may reveal diagnostic markers or therapeutic targets [4, 13, 17]. In addition, miRNA profiles can be used for cancer classification and determination of its stage and progression, as well as for the prognosis and response to treatment [18, 19].

Studies of miRNAs and their targets have shown that the overexpression of both *hsa-miR-21* [20–25] and *hsa-miR-221* [22, 26] participates in the initiation and progression of oral cancer [24, 25, 27]. *Hsa-miR-29c* hiperexpression was associated with the most aggressive and metastatic cases of OSCC [28, 29] and *hsa-miR-135b* differential expression was associated with poorer overall survival of patients, besides being a key regulator in this type of cancer [30].

Furthermore, previous studies from our group showed the presence of field cancerization in gastric cancer by high throughput miRNA sequencing [31]. Among the identified miRNAs, both *hsa-miR-29c* and *hsa-miR-135b* had different expression profiles in tumor and tumor-adjacent tissues in the field cancerization. It also encouraged us to investigate if these two miRNAs play a role in this process in OSCC.

The aim of this study was to characterize the expression profile of *hsa-miR-221*, *hsa-miR-21*, *hsa-miR-135b*, and *hsa-miR-29c* in non-cancerous tissue and oral cancer and to associate them with the field cancerization effect.

## Methods

### Sample and ethical aspects

This study included samples from 47 individuals categorized into three groups: i) tissue samples from oral cancer (*n* = 28); ii) tumor-adjacent tissue samples (*n* = 11); and iii) non-cancerous gingival tissue samples (*n* = 19). The adjacent-tumor samples were 1 cm from the tumor margin. For control group, gingival tissue without pericoronaritis was collected from healthy non-smoke volunteers who underwent extraction of the 3rd molar. Patients with history of head and neck radio or chemotherapy or patients with autoimmune disease were excluded. For control group (iii), gingival tissue without pericoronaritis was collected from healthy non-smoke volunteers who underwent extraction of the 3rd molar.

Samples were obtained from patients treated at the dental clinic of the UFC from 2014 to 2015. The samples were collected in a 2 mL microcentrifuge tube containing RNAlater and stored until RNA extraction. Clinical information, such as age, gender, tumor location, and risk factors (smoke and alcohol intake) were collected. The histologic samples were classified according to World Health Organization into well, moderately and poorly differentiated squamous cell carcinoma [32].

All research procedures were conducted according to the Declaration of Helsinki, the Nuremberg Code and subject to the Regulations on Research Involving Human Subjects (Res. CNS 196/96) of the Brazilian National Health Council, which respects ethical standards and patients’ rights. Data were collected after the patients signed a free and informed consent form. The project was approved by the Human Research Ethics Committee of the Federal University of Ceará (Universidade Federal do Ceará - UFC), under the protocol number 77/09.

### Total RNA extraction, reverse transcription, and quantitative real-time PCR (qRT-PCR)

Total RNA was extracted following the protocol of the High Pure RNA Isolation kit (Roche Applied Science) and quantified using a Qubit®2.0 fluorometer (Life Technologies, Foster City, CA, USA). The total RNA extracted was diluted in diethylpyrocarbonate (DEPC)-treated water to a final concentration of 5 ng/μL and stored at − 80 °C.

Total RNA (5 ng) was used in a reverse transcription reaction using TaqMan MicroRNA Reverse Transcription kit (Applied Biosystems, Foster City, CA, USA), following the manufacturer’s guidelines. The reverse transcription product was subjected to amplification using TaqMan® MicroRNA Assays and Universal Master Mix II (Applied Biosystems, Foster City, CA, USA) in a Rotor-Gene Q (QIAGEN, Venlo, The Netherlands). All reactions were performed in triplicate, and the comparative Ct method was used to analyze the differences in expression in each group. The expression levels of *hsa-miR-221*, *hsa-miR-21*, *hsa-miR-135b*, and *hsa-miR-29c* were normalized by using the endogenous control RNU6B (Applied Biosystems, Foster City, CA).

### In silico prediction of hsa-miR-21, hsa-miR-221, hsa-miR-29c and hsa-miR-135b target genes

In order to identify genes that may be involved in OSCC, we searched for the of ***hsa-miR-21, hsa-miR-221, hsa-miR-29c*** **and** ***hsa-miR-135b*** target genes by using miRecords (http://c1.accurascience.com/miRecords/) (which integrates 11 prediction tools), TargetCompare (http://54.187.40.156:8080/targetcompare/), miRTarBase (http://mirtarbase.mbc.nctu.edu.tw), miRo (http://www.dmi.unict.it/~ferro/) and miRNAMap (http://mirnamap.mbc.nctu.edu.tw). We considered target genes the ones that were observed in no less than 10 tools and have already been validated experimentally.

### Statistical analysis

To examine differences in the expression of *hsa-miR-221*, *hsa-miR-21*, *hsa-miR-135b*, and *hsa-miR-29c* among the cancerous, adjacent, and non-cancerous groups, ΔCt values were used. Normality of the data was evaluated by the Kolmogorov-Smirnov test. One-Way ANOVA or Kruskall-Wallis (for parametric and non-parametric distributions) was used to compare the expression values among the three groups. Tukey’s HSD correction was applied for multiple pairwise comparisons. Differences with a *p*-value < 0.05 were considered to be statistically significant. T-student was used to compare miRNA expression and clinical data. To estimate the sensitivity of the biomarker for distinguishing the groups, Receiver Operating Characteristic (ROC) analysis and the Area Under the Curve (AUC) were used. A Spearman rank correlation was performed to verify if there was a correlation between the expression of *hsa-miR-21* and STAT3 staining scores in OSCC cases. All tests and graphs were done using the statistical package R (www.R-project.org).

### Immunohistochemistry

Immunohistochemical experiments were performed on histological sections “Conclusions” μm thick on previously identified silanized histological slides, following the streptavidin-biotin-peroxidase technique phospho-STAT3 (ThermoFisher®, policlonal), diluted 1:800. The silanized slides were incubated at 65 °C for 1 h, and after this period, deparaffinized in xylene and alcohol gradient. Antigen retrieval was performed in pH 6 buffer, in microwave. After blocking the endogenous peroxidase activity (aqueous solution of H202 3%), the primary antibodies were incubated for 30 min. After washing, the secondary antibody was added (Bond Polymer Refine Detection, DS9800, Leica®), for 10 min followed by streptavidin-biotin-peroxidase complex (10 min), and then revealed with chromogen diaminobenzidine (K3468, DAKO) for 10 min. The slides were counterstained with Harris hematoxylin for 30 s and mounted. As a negative control, the primary antibody was omitted from the reactions, and for a positive control, we used fragment of colon adenocarcinoma.

Digital Images from histologic slides were standardized obtained using the camera (DFC 295) equipped to a light microscope (Leica® DM 200). The procedure consisted of an initial scan of the tumor, using a small increase (40×) to identify areas of higher stain. Then, using a magnification of 200×, color digital images were captured of five aleatory fields. The images were stored in Windows® Bitmap (BMP) format.

A quantification of STAT-positive and negative cells was made by evaluating the stain intensity and if it was nuclear or cytoplasmic (MBF Image J, MacBiophotonics, McMaster University, Hamilton, ON, Canada). The percentage of positive cells was acquired to perform the Label Index (LI). Score 0 was attributed if LI ≤ 10%; score 1 if LI ≤ 11–30%; score 2 if LI ≤ 31–50%; score 3 if LI ≤ 51–60%; score 4 if LI ≤ 71–100%. The intensity of stain was categorized in score 0 (negative), score 1 (mild), score 2 (moderate), score 3 (intense). Finally, the scores obtained from LI and intensity were adding and a total score was acquired. The field was considered positive when total score was 3 or higher [33].

## Results

### Sample characterization

The tumor samples (group i) was composed by moderate (*n* = 25) and well differentiated squamous cell carcinoma (*n* = 3); and controls samples (group iii) were of non-cancerous gingival tissues (*n* = 19). The OSCC (Oral squamous cell carcinoma) sites were tongue (*n* = 10), gingiva (*n* = 8), floor of mouth (*n* = 5), cheek (*n* = 4) and retromolar trigone (*n* = 1). Most volunteers were male (*n* = 18) and the age varied from 62 to 84 (media =62 years) (Table 1).

**Table 1:** The clinicopathologic characteristics in OSCC patients

Clinical Characteristics	Average / N° (Range / %)
Age	62 (62–84)
Histological Classification	
Moderate differentiated	25 (89.3%)
Well differentiated	3 (10.7%)
Gender	
Male	18 (64.3%)
Female	10 (35.7%)
Smoking condition	
Yes	19 (67.8%)
No	9 (32.2%)
Alcohol consumption	
Yes	11 (39.3%)
No	17 (60.7%)
OSCC sites	
Tongue	10 (35.7%)
Gingiva	8 (28.6%)
Floor of mouth	5 (17.8%)
Cheek	4 (14.2%)
Retromolar trigone	1 (3.7%)

Among the evaluating risk factors, 19 were positive for smoking, and 11 were positive to alcohol consumption.

The collected tumor samples (group i) was composed by moderate (*n* = 25) and well differentiated squamous cell carcinoma (*n* = 3). Among the evaluating risk factors, 19 were positive for smoking, and, of whom 11 were positive to too alcohol drinkers consumption. The OSCC sites were, tongue (*n* = 10), gingiva (*n* = 8), floor of mouth (*n* = 5), cheek (*n* = 4) and retromolar trigone (*n* = 1) (Table 1). Most volunteers were male (n = 18) and the age varied from 62 to 84 (media =62 years) (Table 1).

### Expression profile of hsa-miR-221, hsa-miR-21, hsa-miR-135b, and hsa-miR-29c in OSCC

The normalized expression data of *hsa-miR-221*, *hsa-miR-135b*, and *hsa-miR-29c* followed a normal Gaussian distribution, and ANOVA indicated significant differences between the non-cancerous (gingival samples from healthy volunteers) and cancerous groups as well as between the non-cancerous and adjacent groups. Pairwise comparisons revealed that the expression levels of these three miRNAs were significantly increased in OSCC and adjacent tissues (Table 2).

**Table 2:** Comparison of expression data and significance levels for miRNAs among the different types of tissue

MicroRNA	Normal x Cancer (*P* value)	Normal x Adjacent (*P* value)	Adjacent x Cancer (*P* value)
*hsa-miR-221*	0.0001	0.008	1
*hsa-miR-135b*	8.4E^−7^	9.7E^−6^	1
*hsa-miR-29c*	0.04	0.005	0.49
*hsa-miR-21*	7. 6E^−8^	7.3E^−6^	0.63

**Fig. 1:**
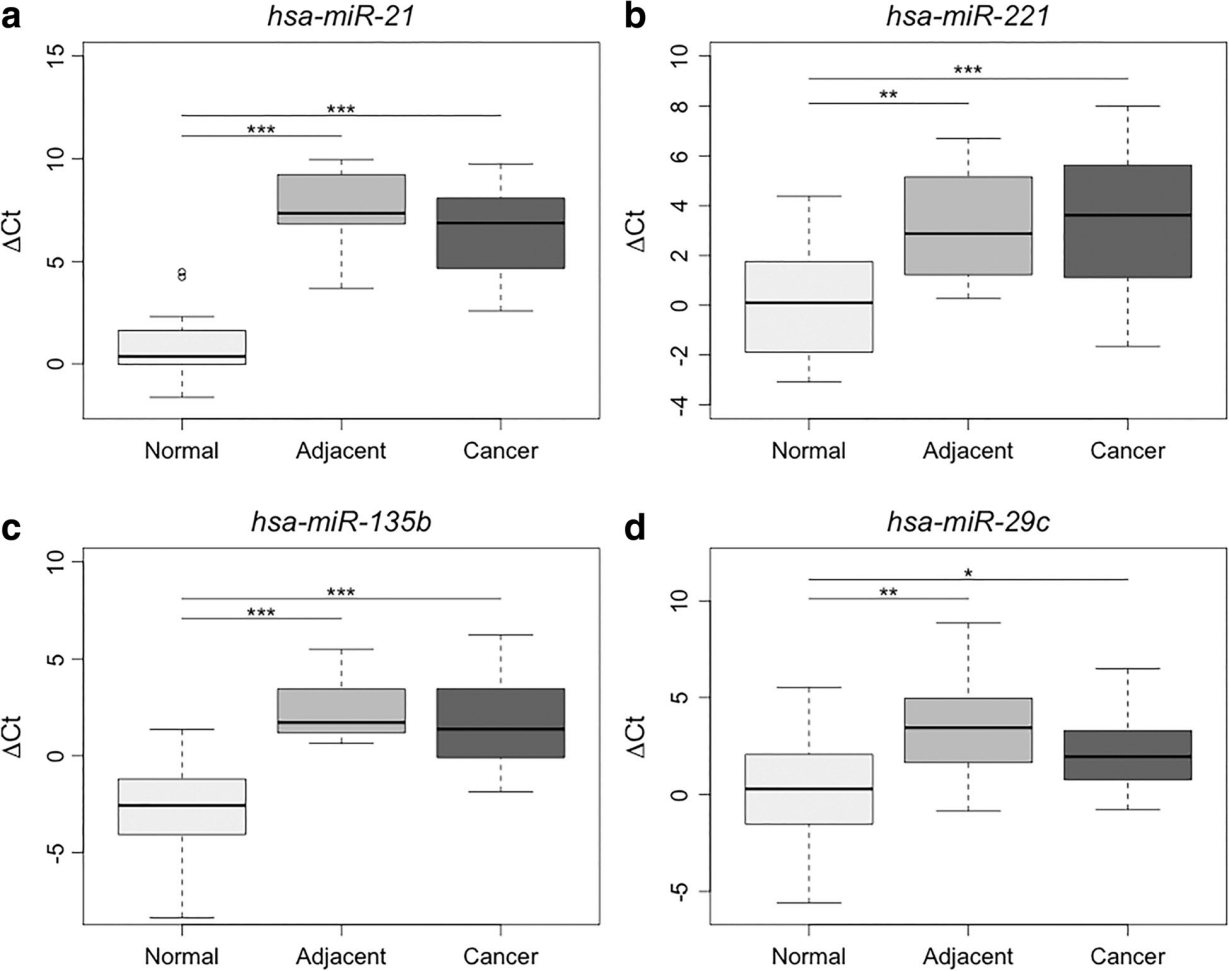
Normalized expression values (ΔCt) of *hsa-miR-221*, *hsa-miR-21*, *hsa-miR-135b*, and *hsa-miR-29c* in non-cancerous, cancer-adjacent, and cancer tissue samples (**a**-**d**)

The expression profile of *hsa-miR-21* was the only variable that did not follow a normal Gaussian distribution, and thus, we used the Mann-Whitney test to analyze this miRNA. The expression of *hsa-miR-21* was significantly higher in OSCC tissue and tumor-adjacent tissue than in non-cancerous tissue (Table 2). In addition, a comparison of expression profiles between tumor-adjacent tissue and OSCC showed no significant difference (Table 2; Fig. 1).

### The expression profiles of *hsa-miR-221*, *hsa-miR-21*, *hsa-miR-135b*, and *hsa-miR-29c* as biomarkers of carcinogenesis in OSCC

To determine whether the expression profiles of *hsa-miR-21*, *hsa-miR-221*, *hsa-miR-135b* and *hsa-miR-29c* could be used as risk factors of carcinogenesis, ROC curves were analyzed, and the discriminatory accuracy was calculated for AUC values. Our results showed that the expression levels of these miRNAs could differentiate the non-cancerous tissue group from the cancer and cancer-adjacent groups.

**Fig. 2:**
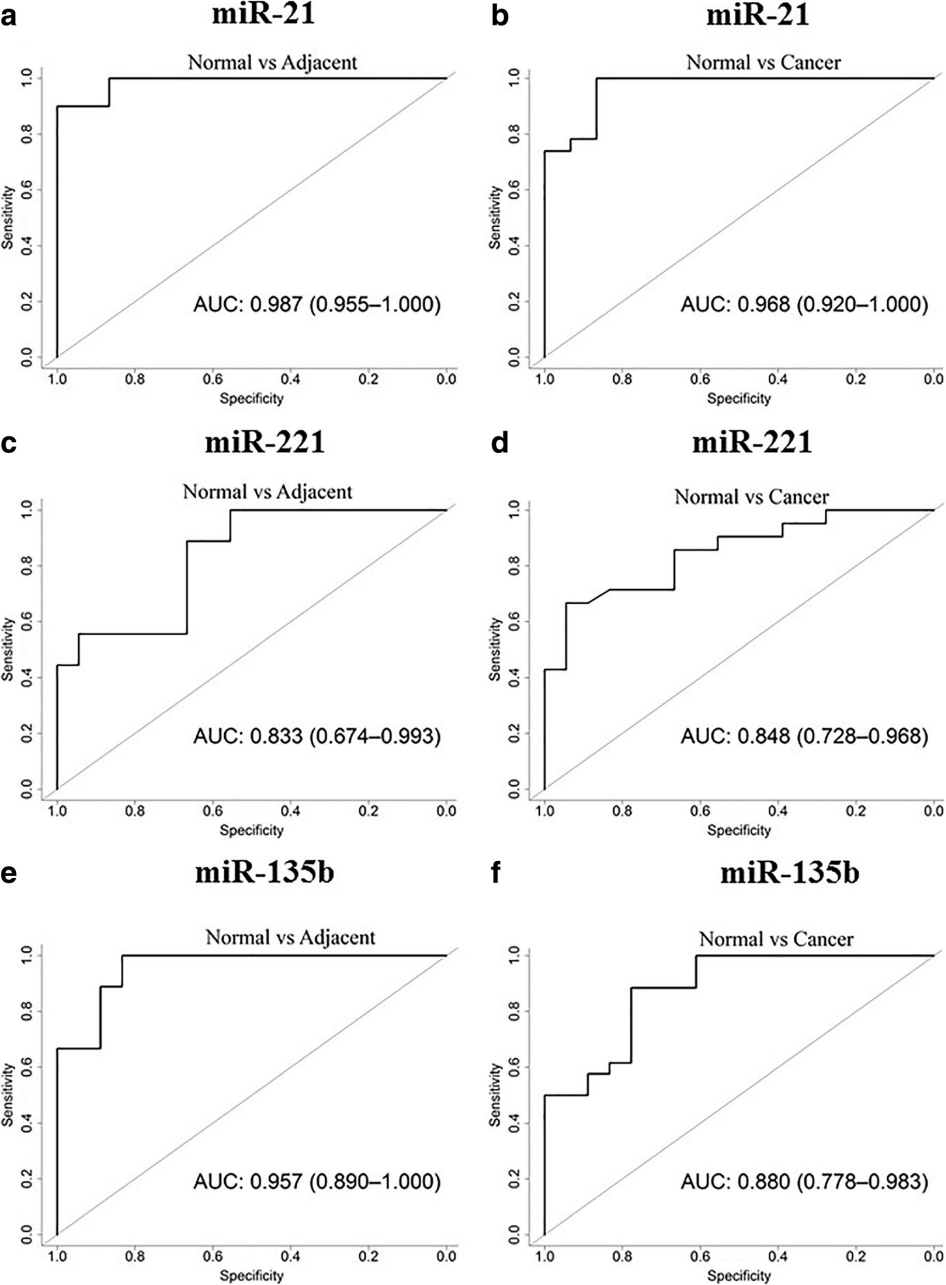
Receiver operating characteristic curves of *hsa-miR-21* (**a**), *hsa-miR-221* (**c**), *hsa-miR-135b* (**e**) and *hsa-miR-29c* (**g**) differentiating OSCC patients from normal controls and those differentiating between normal controls and adjacent tissue using *hsa-miR-21* (**b**), *hsa-miR-221* (**d**), *hsa-miR-135b* (**f**) and *hsa-miR-29c* (**h**)

The expression level of *hsa-miR-21* showed higher discriminatory accuracy between the groups with and without cancerous tissue, exhibiting an accuracy of 96.8% [AUC = 0.968; 95% CI: 0.920–1.00]. Similarly, the non-cancerous and adjacent groups had an accuracy of 98.7% [AUC = 0.987; 95% CI: 0.987–1.00] (Fig. 2).

*Hsa-miR-221* showed a better discriminatory accuracy between the groups with and without cancerous tissue with an AUC of 0.848 (95% CI: 0.728–0.968), followed by the discriminatory power for differentiating between the non-cancerous and adjacent tissue groups (AUC = 0.833; 95% CI: 0.674–0.993) (Fig. 2).

The discriminatory power of *hsa-miR-135b* for differentiating the groups with CCEO and normal tissue resulted in an AUC of 0.880 (95% CI: 0.778–0.983), whereas for the non-cancerous and tumor-adjacent tissue groups, the AUC was 0.957 (95% CI: 0.890–1.00) (Fig. 2).

The discriminatory power of *hsa-miR-29c* for differentiating between the groups with and without oral cancer resulted in an AUC of 0.880 (95% CI: 0.778–0.983), whereas for the non-cancerous and tumor-adjacent tissue groups, the AUC was 0.957 (95% CI: 0.890–1.00) (Fig. 2).

### In silico prediction of hsa-miR-21, hsa-miR-221, hsa-miR-29c and hsa-miR-135b target genes

We identified that miRNAs *hsa-miR-21, hsa-miR-221* and *hsa-miR-29c* share two target genes that have been demonstrated to participate in oral carcinogenesis – phosphatase and tensin homolog (*PTEN*) and *DICER1*. *hsa-miR-21* and *hsa-miR-135b* share the target gene Adenomatous Polyposis Coli (*APC*). The *STAT3* gene was identified as *hsa-miR-21* target.

### Immunohistochemistry

In 88% of OSCC, STAT3 was positive and the scores ranged between 2 and 6. The cytoplasmic stain was more frequent than nuclear and the intensity of stain varied from mild (89%) to moderate (11%). Comparison between STAT immunostaning and clinical variables showed that cytoplasmic stain was more intense in moderately differentiated OSCC and that males presented a higher final score.

**Fig. 3:**
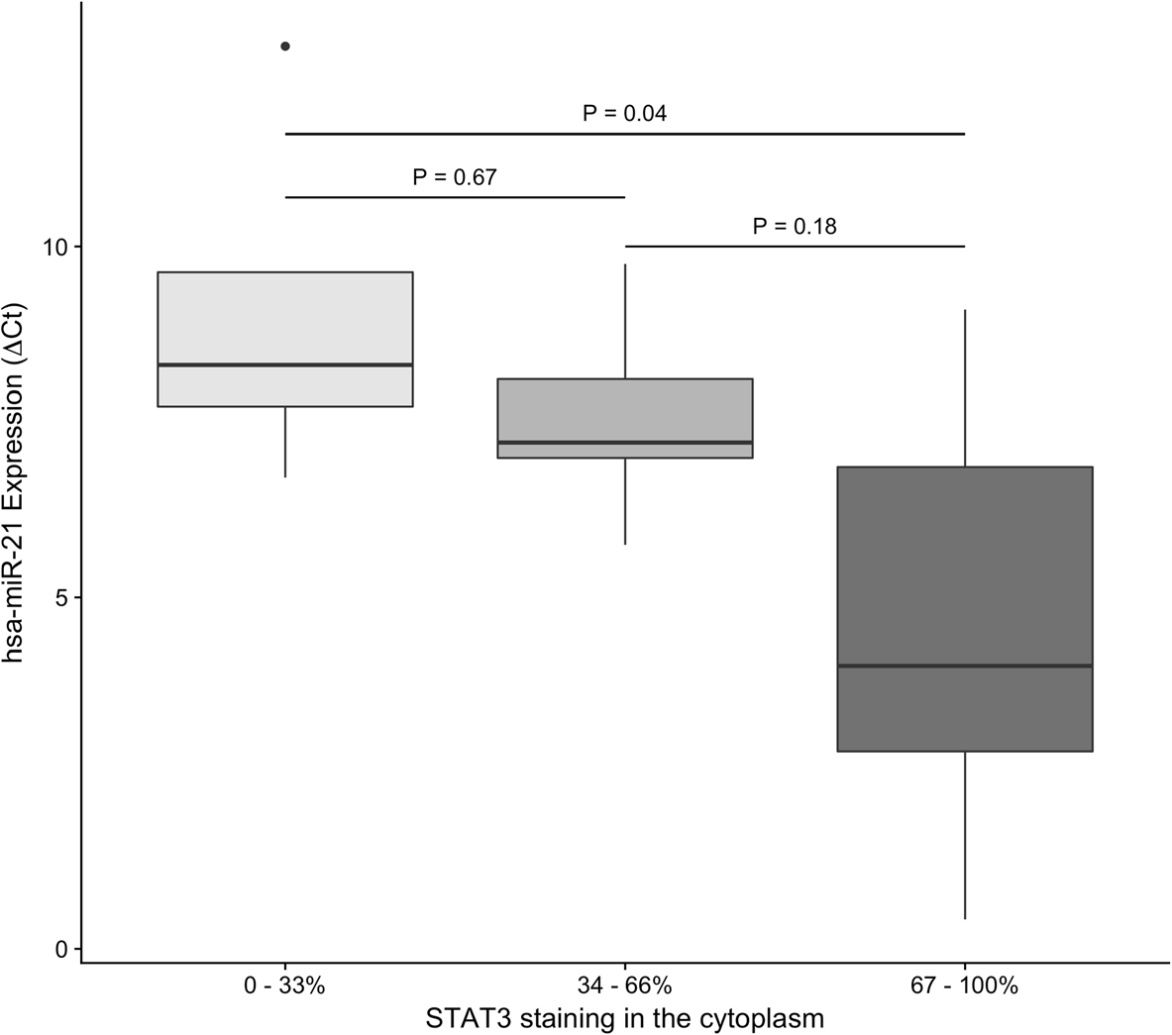
*Hsa-miR-21* expression in OSCC patients and its correlation with STAT3

We also compared the expression of *hsa-miR-21* with the percentage of STAT3 staining in the cytoplasm of OSCC cases. We observed that was a difference in *hsa-miR-21* expression along the STAT3 staining scores in the cytoplasm (ANOVA, *P* = 0.036). The expression of this miRNA was lower in the group that had the highest percentage of STAT3 staining (Fig. 3, *P* = 0.04). Additionally, we performed a Spearman rank correlation and found a negative correlation between *hsa-miR-21* and STAT3 expressions (*r* = − 0.52, *P* = 0.025).

## Discussion

Most studies in OSCC compare tumor tissue samples with tumor-adjacent tissue samples for investigating genetic and epigenetic markers [17, 20, 21, 25, 27, 28]. Using this approach, researchers consider the tissue surrounding the the tumor tissue as a non-cancerous sample [20, 21, 25]. In this study, we compared three sample groups: i) tissue samples from oral cancer (*n* = 28); ii) tumor-adjacent tissue samples (*n* = 11); and iii) non-cancerous gingival tissue samples.

Expression levels of *hsa-miR-21*, *hsa-miR-221*, *hsa-miR-29c* and *hsa-miR-135b* showed significant differences between non-cancerous and adjacent to the tumour tissues, and demonstrated no significant difference between cancer and tumor-adjacent tissues (*P* = 0.63; *P* = 1; *P* = 0.49 and *P* = 1, respectively). These four miRNAs suggest the occurrence of a field cancerization effect in OSCC, and their dysregulation may provide an environment permissive for a cascade of events that may promote oral carcinogenesis.

*hsa-miR-21* is an oncomiR that is overexpressed in several types of carcinomas, including colorectal cancer [34], esophageal cancer [35], hepatocellular cancer [36] and OSCC [20–25]. Blocking *hsa-miR-21* expression inhibits or reduces cell growth and proliferation both in vitro and in vivo and induces apoptosis [24, 35]. Several studies have demonstrated the overexpression of *hsa-miR-21* in OSCC [21, 25, 37, 38]; however, most of these studies used the tumor-adjacent for comparative analysis. Our results also show the 4.57 fold overexpression of *hsa-miR-21* in tumor-adjacent tissue compared to the non-cancerous tissue samples (*P* = 7.3E^− 6^), indicating that the area surrounding the tumor already presents an altered expression profile for this miRNA and thus cannot be considered normal.

The *hsa-miR-221* expression profiles in head and neck squamous cell carcinomas (HNSCC) have shown its relationship with oncogenesis and cell invasion. In addition, studies showed overexpression of *hsa-miR-221* increased proliferation, cell growth, and migration, and thus, this miRNA is involved in the tumorigenesis of OSCC. Consequently, this marker could be useful for defining strategies for the prevention and treatment of HNSCC [22, 26]. Our results also showed the involvement of *hsa-miR-221* in the carcinogenesis of OSCC, once it was overexpressed in both tumor (29.3 fold) and tumor-adjacent (2.04 fold) samples (*P* = 0.0001 and *P* = 0.008, respectively).

*hsa-miR-135b* is overexpressed in cancers such as colorectal cancer [34], lung cancer [39], cervical cancer [40] and gastric cancer [14]. In lung cancer, *hsa-miR-135b* acts as an oncomiR and promotes tumor growth and cell invasion, and contributes to angiogenesis and metastasis; thus, it seems to play an important role in multiple cancer development processes [39, 41]. Few studies have shown the expression of *hsa-miR-135b* in OSCC, therefore, and this study does corroborate the previous works.. We found the expression of *hsa-miR-135b* to be upregulated in both tumor (7.93 fold) and tumor-adjacent (7.96 fold) tissues when compared to normal tissues (*P* = 8.4E^− 7^ and *P* = 9.7E^− 6^, respectively).

*hsa-miR-29c* acts as a tumor-suppressor miRNA (TS-miR) due to its reduced expression in some cancers, such as gastric cancer [14], liver cancer [42] and hepatocellular cancer [43]. However, our results showed the overexpression of *hsa-miR-29c* in OSCC (*P* = 0.04; 2.39 fold), which corroborates the results of a previous study [28].

Furthermore, the miRNAs *hsa-miR-21*, *hsa-miR-221* and *hsa-miR-29c* share two target genes that have been demonstrated to participate in oral carcinogenesis, Phosphatase and tensin homolog (*PTEN*) and *DICER1* [44–47].

*PTEN* functions as a tumor suppressor by negatively regulating the PI3K/Akt signaling pathway, which is involved in multiple biological processes, including cellular apoptosis, cell cycle regulation, survival and proliferation [48, 49]. The aberrant activation of the PI3K/Akt signaling pathway has a significant role in tumorigenesis and tumor metastasis [49, 50]. In OSCC, downregulation of *PTEN* is correlated with the stage of carcinoma differentiation, cell proliferation, invasion and indicate a potential therapy for OSCC [45, 50, 51]. Since *PTEN* is a common target of miRNAs *hsa-miR-21*, *− 221* and *-29c*, its downregulation in OSCC may be due the excessive regulatory activity from these three overexpressed miRNAs.

Dicer is an endoribonuclease coded by *DICER1* gene, that plays an essential role by regulating the miRNA biogenesis [52]. In this endoribonuclease there are two RNase III domains, an intramolecular dimer that can cleave the pre-miRNA hairpin to generate mature miRNAs [53, 54]. Therefore, *DICER1* is one of the most important components involved in miRNA biogenesis and its expression level seemed to correlate with tumor initiation, progression and patients’ prognosis [54–56]. In OSCC, a low Dicer expression may influence the pathogenesis of oral cancer cells and was significantly correlated with the pathological response to chemoradiotherapy. Furthermore, Dicer was suggested as a potential biomarker for predicting the clinical response and a therapeutic target for OSCCs [57–59].

Studies showed that the overexpression of *hsa-miR-21* or *hsa-miR-135b* leads to a downregulated expression of Adenomatous Polyposis Coli (*APC*) gene [60–62]. *APC* encodes a tumor suppressor protein that negatively regulates the Wnt signaling pathway. Consequently, inactivation of the *APC* gene or activation of the WNT-1 pathway causes the nuclear accumulation of β-catenin, hence it seems to lead to deregulated cell adhesion and other processes such as cell migration, and apoptosis [63, 64]. Among the alterations in the *APC* expression, the epigenetic modifications cause a downregulation in its expression in OSCC leading to a blockage of tumor suppressor action and a progress of tumorigenesis [64, 65].

Furthermore, according to Strzelczyk et al. [65] *APC* expression in tumor and tumor-adjacent from patients with OSCC had similar levels, corroborating the field cancerization effect hypothesis. The authors suggested that the cancer field effect should be considered in diagnosis and treatment of cancers, once the remaining field after a surgery may pose an increased risk of cancer development. Thus, molecular analysis on tumor-adjacent tissue and additional research regarding their assessment are necessary and fundamental.

The STAT family of transcription factors are the principal signaling proteins of cytokines, which mediates cell communication and wide range of biological responses. STAT3 is vital in tumorigenesis and cancer-induced immunosuppression [66–68] and its persistent activation has been observed in several cancers [38, 66, 67]. The activation of STAT family and the control of aberrantly expressed miRNAs seems in the most basic mechanisms of OSCC [38, 69, 70]. Furthermore, STAT3 activates *miR-21* to promote cancer cell growth [38, 71]. In our study, we compared the expression of *hsa-miR-21* with the percentage of STAT3 staining in the cytoplasm of OSCC and demonstrated a negative correlation between these two variables. This evidence suggests that STAT3 is a target gene and it is regulated by *hsa-miR-21*, even though Zhou et al., 2014 [38] showed that cytoplasmic *miR-21* and STAT3 were both highly expressed in poorly differentiated OSCC tissue samples when compared to highly differentiated samples.

## Conclusions

Analyzing the expression profiles of *hsa-miR-21*, *hsa-miR-221*, *hsa-miR-135b* and *hsa-miR-29c* in non-cancerous, tumor-adjacent and OSCC tissue samples, we observed evidences of the existence of the field cancerization effect in oral carcinogenesis via an epigenetic approach. Thus, the studied miRNAs have the potential of representing biomarkers for detecting field cancerization. In addition, because of their reported function, these miRNAs are likely to be involved in pathogenic processes associated with OSCC development.

## Abbreviations

HNSCC: head and neck squamous cell carcinomas
miRNAs: microRNAs
OSCC: Oral squamous cell carcinoma
